# Cobalt-loaded cherry core biochar composite as an effective heterogeneous persulfate catalyst for bisphenol A degradation†

**DOI:** 10.1039/d1ra09236g

**Published:** 2022-03-04

**Authors:** Li Li, Yuanyuan Zhang, Shuangshuang Yang, Shengxiao Zhang, Qiang Xu, Pinzhu Chen, Yaxuan Du, Yuxin Xing

**Affiliations:** School of Chemistry and Materials Science, Collaborative Innovation Center of Shandong Province for High Performance Fibers and Their Composites, Ludong University Yantai 264025 Shandong province China beijingzsx@163.com +086 0535-6695905 +086 0535-6696162; Environmental Monitor Station of Yantai No. 118, Qingnian South Road Yantai 264000 Shandong province China

## Abstract

Persulfate (PS)-based advanced oxidation processes have drawn tremendous attention for the degradation of recalcitrant pollutants, and cobalt composites are effective for PS activation to generate reactive species. In this study, composites of cobalt species loaded on cherry core-derived biochar (Co/C) were prepared with a one-step pyrolysis method. The Co/C catalyst was applied as a catalyst for PS activation to degrade bisphenol A (BPA). Factors influencing the degradation efficiency were examined, including the ratio of raw materials, Co/C and PS dosages, temperature, and solution pH. The Co/C catalyst prepared when the ratio of raw material was 1 : 1 (Co/C-50) could efficiently activate both peroxymonosulfate (PMS) and peroxydisulfate (PDS). When the initial concentration of BPA was 20 mg L^−1^, complete removal of BPA was achieved in the Co/C-50-PMS and Co/C-50-PDS systems within 8 min and 10 min, respectively. More than 70% of BPA could be mineralized in the Co/C-50-PS system. The free radical quenching experiments demonstrated that in the Co/C-50-PS system, the degradation of BPA was achieved through free radical, surface-bound free radical, and non-free radical pathways. The successful preparation of the Co/C-50-PS catalyst broadens the application of cobalt-based carbon materials in the activation of PS to remove organic pollutants.

## Abstract 2

Co/C composites were prepared with a one-step pyrolysis method for the activation of persulfate to degrade organic pollutants.

## Graphical abstract

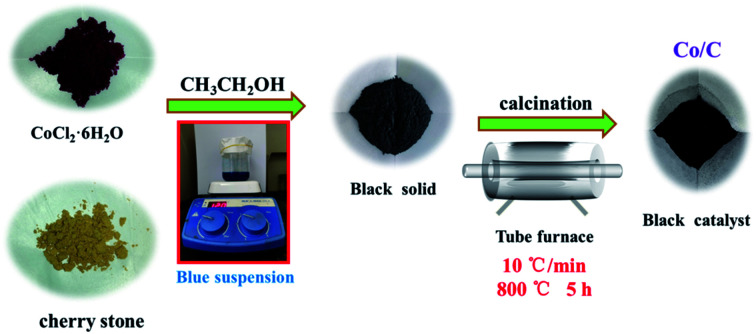

## 1. Introduction

Bisphenol A (BPA) is one of the most common incretion disrupting compounds that bind to estrogen receptors in the body and interfere with the synthesis, metabolism and activity of normal oestrin,^1^ and it is extensively used in industrial processes as a plasticizer, such as in plastic manufacturing, adhesives, elasticizers, and hardeners.^2^ Due to its wide application, it is frequently detected in drinking water, surface water, wastewater and even treatment wastewater. These pollutants are toxic, carcinogenic, teratogenic and mutagenic.^3–7^ For example, BPA can increase the risk of developing ovarian cancer, prostate cancer, asthma and leukemia.^8^ The physical and chemical properties of BPA are stable, and it is difficult to remove BPA effectively by biodegradation. Therefore, there is an urgent need to develop effective degradation technology for BPA removal.

A variety of remediation technologies have been developed for BPA degradation, among which advanced oxidation processes (AOPs) have been paid increasing attention due to their easy operation, strong oxidation degradation ability, and lower secondary pollution.^9^ Advanced oxidation technology based on the sulfate radical 
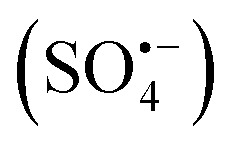
 is a new type of technology developed in recent years to treat refractory organic pollutants.^10^
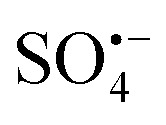
 is mainly produced by the activation of persulfates, which include peroxymonosulfate (PMS) and peroxydisulfate (PDS), both of which have unstable O–O bonds and are prone to external electron fracture.^11^ The standard redox potential of 
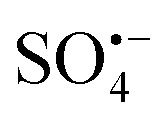
 (*E*^*θ*^ = 2.5–3.1 V) is higher than that of ˙OH (*E*^*θ*^ = 1.8–2.7 V); therefore, 
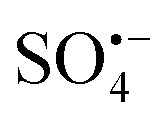
 is a strong oxidant, and the half-life of 
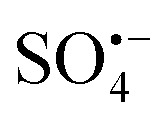
 (about 30–40 μs) is much higher than that of ˙OH (less than 1 μs), which indicates that 
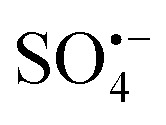
 is more stable and has a longer contact time with potential pollutants compared to ˙OH.^12^ Moreover, 
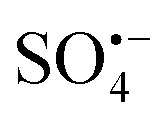
 can maintain high activity in a wide range of solution pH values, especially in neutral or alkaline conditions, showing higher activity than ˙OH.^13^ Therefore, compared with other traditional water treatment technologies, AOPs based on 
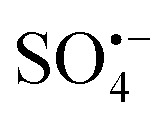
 (S-AOPs) have the advantages of high efficiency, rapidity, thoroughness, low selectivity, and mild reaction conditions,^14^ and have wide application prospects in the field of environmental pollution remediation and treatment.


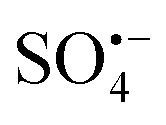
 was produced mainly by activating persulfate, and common activation methods include heat,^15^ microwave,^16,17^ ultrasound,^18^ UV radiation,^19,20^ transition metal,^21,22^ carbon composites,^23,24^ and alkali.^25^ Transition metal activation has the advantages of low energy consumption, high efficiency, and mild reaction conditions. Some metal catalysts (such as cobalt, copper, iron, and manganese) been demonstrated to be effective in activating persulfate.^26–28^ Among them, cobalt is considered to be the most effective non-noble metal catalyst for persulfate activation. However, homogeneous cobalt ions added to a solution are not easy to recover, leading to secondary contamination. Therefore, heterogeneous Co-based catalysts gradually substituted with homogeneous Co ions have been widely applied to activate PS. To date, scholars have studied a variety of heterogeneous cobalt-based catalysts, mainly including cobalt oxide (CoO, CoO_2_, Co_2_O_3_, CoO(OH), Co_3_O_4_), spinel cobalt ferrite (CoFe_2_O_4_) and supported cobalt catalysts (supported substrates include MO_*X*_ metal oxides, molecular sieves, carbon materials, and MOFs).^29^ Biochar is a new kind of low-cost carbonaceous material that is produced by low-cost waste biomass residues. Due to its high specific surface area, excellent electronic conduction properties and good stability, biochar is a promising candidate for loading of metal nanoparticles. Tian *et al.*^30^ impregnated corn straw by hydrothermal carbonization in CoCl_2_ solution, and they successfully loaded Co_3_O_4_ nanoparticles on the surface of biochar to manufacture a Co-BC composite for activating PMS to degrade sulfamethazine in aqueous solution.

In this study, Co/C composites were manufactured by using cherry core powder and CoCl_2_·6H_2_O to activate PS to degrade pollutants, which provides an available method for large-scale production and application of environmental materials in the AOPs field. Cherry core biochar composites supported with Co species were synthesized and used as catalysts for activated persulfate to degrade BPA. The influencing factors of the degradation of BPA by the Co–C composites activating PS were investigated, and the reuse and practical application of the materials were examined.

## 2. Materials and methods

### 2.1 Chemicals and reagents

The chemicals and reagents are shown in Text S1.†

### 2.2 Synthesis of Co/C

CoCl_2_·6H_2_O and cherry kernel powder were scattered in 50 mL ethanol and heated to 120 °C with magnetic stirring until dried. After steam-drying, the solid matter was removed and ground in a mortar. It was calcined in a tubular furnace under argon atmosphere for 5 h at 800 °C. The amounts of CoCl_2_·6H_2_O and cherry stone were changed to make Co/C materials with different cobalt contents; they were labeled as C, Co/C-10, Co/C-20, Co/C-30, Co/C-40, Co/C-50, Co/C-60, Co/C-70, Co/C-80 and Co/C-90, respectively. The specific preparation method is presented in Table S1.†

### 2.3 Material characterization

The characterization methods of the materials are shown in Text S2.†

### 2.4 Degradation experiments

The catalytic degradation of BPA was carried out in a glass conical flask. Certain amounts of Co/C-50 and persulfate were added to 100 mL BPA solution (20 mg L^−1^) and oscillated for 30 min in a water bath thermostatic oscillator at 30 °C. The initial pH was regulated with HCl and NaOH solution. Within a certain time interval, 3 mL solution was taken out and mixed with 3 mL methanol to quench the reaction. The mixture was centrifuged, and the supernatant was taken for analysis. The concentration of BPA was analyzed by high performance liquid chromatography (HPLC, UltiMate 3000, US). The mobile phase consisted of methanol and 0.5% phosphoric acid (volume ratio: 80 : 20), the flow rate was 1 mL min^−1^, and the column temperature was 30 °C. In the recyclability test, Co/C-50 was separated and recovered with a magnet (Text S3 and Fig. S1†), and then simple SnCl_2_ washing and water washing regeneration were carried out for the next experiment. A TOC-L analyzer (Shimadzu, Japan) was used to determine the total organic carbon (TOC) of the aqueous solutions.

## 3. Results and discussion

### 3.1 Characterization of Co/C

#### 3.1.1 XRD and Raman spectral characterization

**Fig. 1:**
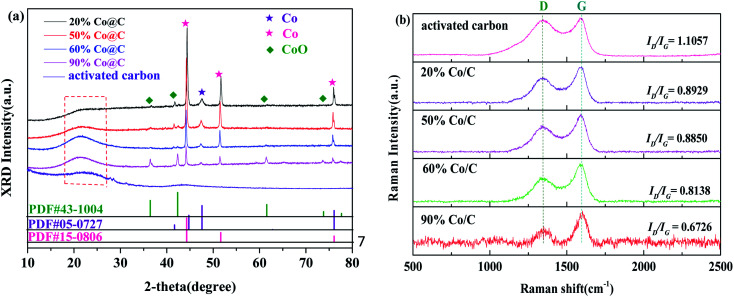
XRD patterns (a) and Raman spectra (b) of the five different samples.

Five catalysts (activated carbon, Co/C-20, Co/C-50, Co/C-60 and Co/C-90) were characterized by XRD and Raman spectra. The diffraction peaks of Co^0^ (111), (101), (200) and (220) were at 44.22°, 47.57°, 51.52° and 75.85°, respectively (JCPDS no.15-0806 and no.05-0727). This indicated that Co^0^ nanocrystals appeared in the catalyst (Fig. 1a), and their formation was due to the reduction of Co^2+^ by the biomass activated carbon during calcination. With the increase of Co content, the diffraction peak of the carbon (002) crystal plane became smaller and narrower; this indicated that the addition of Co increased the graphitization degree of activated carbon, and the graphitization degree increased with the increase of the Co content. The enhanced graphitization degree was favorable for electron transfer.^31^ These results indicated that the Co/C catalysts were more conducive to PS activation than activated carbon. Meanwhile, the four crystal faces of CoO (111), (200), (220) and (311) at 36.50°, 42.40°, 61.52° and 73.70°, respectively (JCPDS no. 43-1004), were also detected in the XRD patterns. In the Raman spectrograms (Fig. 1b), D and G peaks appeared in all five materials, and the *I*_D_/*I*_G_ value was also used to measure the graphitization degree of the five catalysts. As can be seen from Fig. 1b, the *I*_D_/*I*_G_ value decreases with the increase of Co content. This indicates that the graphitization degree of the material increases with the increase of Co content, which is in agreement with the XRD results.

#### 3.1.2 FT-IR characterization

The four Co/C materials were characterized by Fourier transform infrared spectroscopy (FT-IR). As shown in Fig. S2,† the strong and wide absorption peak at 3432 cm^−1^ was the stretching vibration peak of O–H; the absorption peaks near 2922 cm^−1^ and 2853 cm^−1^ were the stretching vibration peaks of C–H; at 1631 cm^−1^; the slightly stronger absorption peaks were C=C and C=O stretching vibration peaks; the peak at 1384 cm^−1^ corresponded to the characteristic peak of O–C=O; and the stretching vibration peaks of C–C and C–O appeared at 1114 cm^−1^. These findings demonstrated that the Co/C catalysts had abundant oxygen-containing and carbon-containing functional groups. The Co–O peaks near 660 cm^−1^ and 570 cm^−1^ indicated that the Co/C catalysts contained CoO,^32^ which was consistent with the XRD results.

#### 3.1.3 XPS characterization

**Fig. 2:**
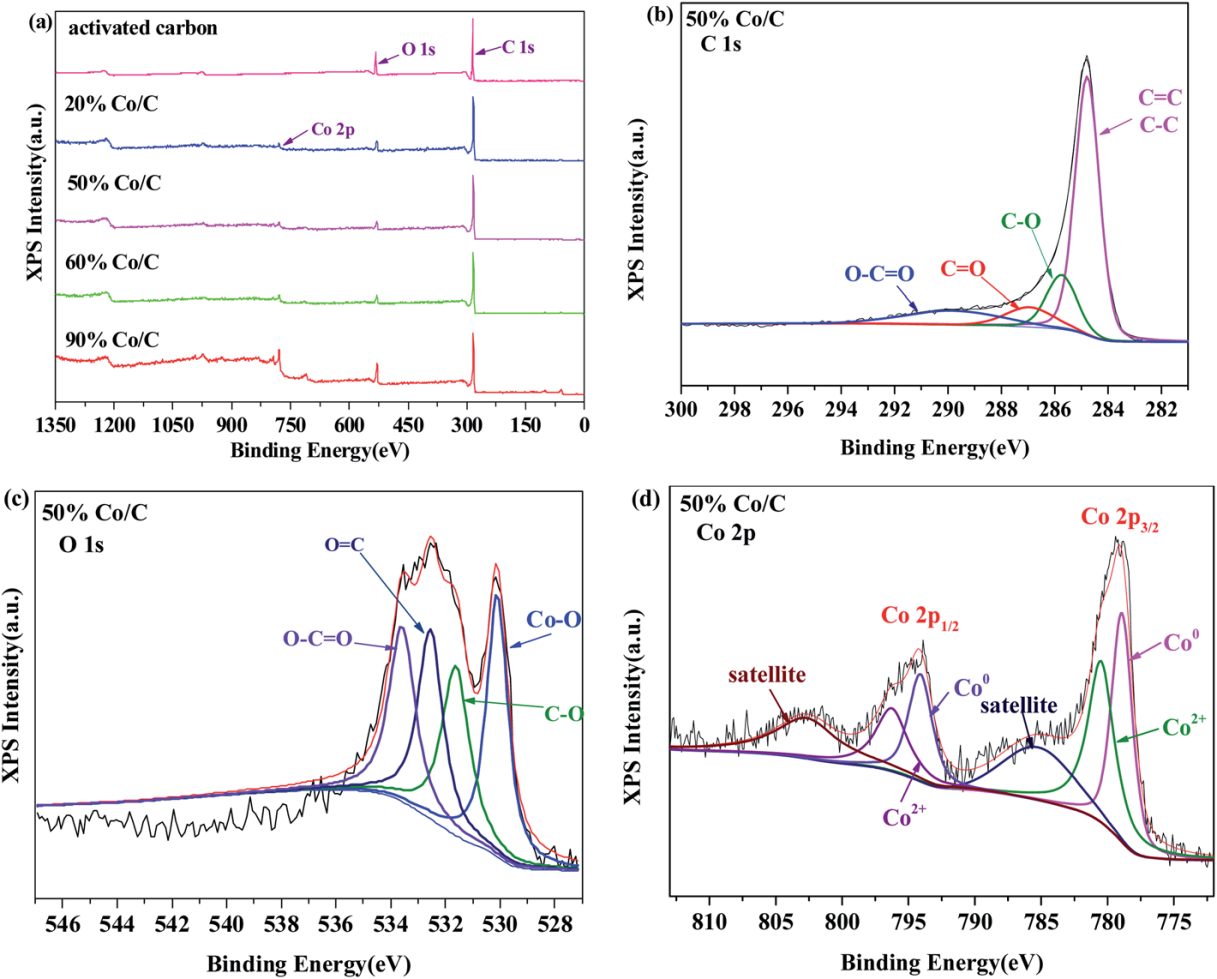
XPS spectra of the as-prepared catalysts: (a) survey, (b) C 1s, (c) O 1s, (d) Co 2p.

The valence of Co/C was characterized by XPS. In the XPS diagrams of Co/C-20, Co/C-50, Co/C-60 and Co/C-90 (Fig. 2a), the photoelectron characteristic peaks of O 1s and C 1s in activated carbon were observed, and a peak of Co 2p was detected at 780.34 eV, indicating that Co element was successfully loaded onto the carbon substrate. The C 1s fine spectrum of Co/C-50 (Fig. 2b and c) showed that the main forms of C included C=C/C–C (284.5 eV), C–O (286.1 eV), C=O (286.56 eV), and O–C=O (289.2 eV),^33,34^ and O 1s exhibited not only O–C (531.3 eV), O=C (532.5 eV), and O–C=O (533.5 eV), but also the peak of the Co–O bond at 530.1 eV as the main oxygen-containing functional groups.^35^ Moreover, we decomposed the peak of Co 2p in detail. As shown in Fig. 2d, the Co 2p spectrum consisted of two spin orbit peaks and two satellite peaks of Co 2p_1/2_ and Co 2p_3/2_, and the peaks of Co^0^ (778.8 eV, 794.1 eV) and Co^2+^ (780.5 eV, 796.5 eV) were found in Co 2p_1/2_ and Co 2p_3/2_,^36^ confirming the existence of Co and CoO crystals; this was consistent with the XRD results.

#### 3.1.4 HR-TEM characterization

**Fig. 3:**
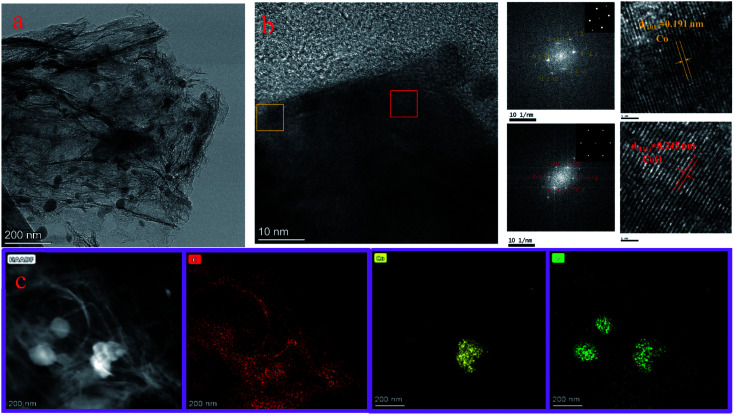
(a) TEM image, (b) HR-TEM image, and (c) EDS mapping of Co/C-50.

The morphologies and lattice structures of the Co/C-50 catalysts were analyzed by HR-TEM. The TEM images (Fig. 3a) exhibited that the biomass activated carbon was distributed in sheets, and it had a very thin layered structure; moreover, some areas showed multi-layer stacking. Spherical CoO_*x*_ nanoparticles (10–50 nm) were dispersed on the carbon substrate, and no isolated CoO_*x*_ nanoparticles appeared outside the carbon substrate, which was beneficial to the dispersion and stability of the CoO_*x*_ nanoparticles. HR-TEM images (Fig. 3b) were used to clarify the crystal types and crystal faces of CoO_*x*_. The crystal plane spacings of Co^0^ (101) and CoO (111) were 0.191 nm and 0.245 nm, respectively. Selected area electron diffraction (SAED) showed the single crystal characteristic of the Co^0^ and CoO nanoparticles. The results indicated that both Co^0^ and CoO existed in Co/C-50, which was consistent with the results of XPS and XRD. The Co^0^ and CoO lattice structures were arranged in an orderly manner and formed a good combination with the carbon substrate. Element mapping images (Fig. 3c) showed that Co/C-50 was composed of C, O and Co, and Co and O were distributed on the carbon substrate. ICP-MS (NexION 300X) was used to determine the contents of cobalt in the catalysts (Table S2†), and the results exhibited the same trend as the ratios of raw materials.

### 3.2 Catalytic degradation experiment

#### 3.2.1 Effects of different mass ratios of Co/C

The performance of Co/C with different mass ratios to activate PS and degrade BPA was investigated. The pseudo-first-order kinetics model was used to fit the degradation data, and the equation was as follows (eqn (1)):

1 ln(*c*_*t*_/*c*_0_) = −*k*_1_*t*

where *k*_1_ (min^−1^) is the kinetics rate constant, *t* (min) is the reaction time, and *c*_*t*_ and *c*_0_ (mg L^−1^) are the concentrations of BPA at times *t* and 0, respectively.

**Fig. 4:**
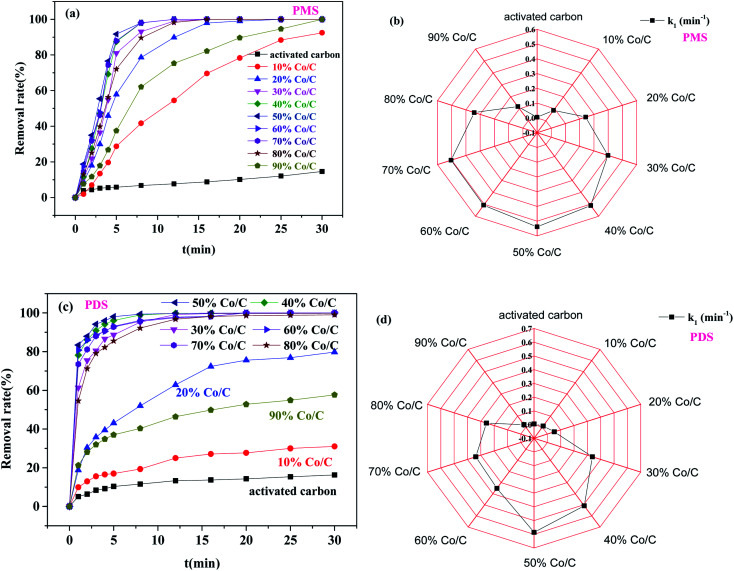
The effects of different mass ratios of Co/C on BPA degradation: removal efficiency of BPA with (a) PMS and (c) PDS; the pseudo-first-order kinetic constants of (b) PMS and (d) PDS (PMS system: *m*_catalyst_ = 0.0050 g, [PMS] = 0.25 g L^−1^; PDS system: *m*_catalyst_ = 0.04 g, [PDS] = 1.0 g L^−1^, [BPA] = 20 mg L^−1^, *T* = 30 °C).

Compared with the other Co supported catalysts, activated carbon-activated PS had the lowest degradation efficiency of BPA (Fig. 4). The load of Co on the activated carbon could remarkably improve the catalytic activity. In addition, as the content of Co increased from 0% to 50%, the degradation efficiency of BPA was enhanced. However, when the Co content was over 50%, the degradation efficiency of BPA was attenuated with the increase of the Co content. The *k*_1_ values showed a similar trend. When the Co content reached 50%, the maximum value of *k*_1_ occurred (PMS: 0.517 min^−1^, PDS: 0.586 min^−1^). This was mainly due to the limited active sites of the catalyst when the Co content was too low. With the increase of Co content, more active sites could be provided for persulfate activation. However, when the Co content outstripped 50%, excessive metal ions gathered in the catalytic center, resulting in decreases of the catalytic activity^37^ and degradation efficiency. This indicated that the appropriate proportion of Co loading could promote the catalytic activity of Co/C.^33^ Because Co/C-50 showed the best catalytic activity and had a high specific surface area (262.5032 m^2^ g^−1^) (Text S4 and Fig. S3†), the Co/C-50 catalyst was selected for investigation in the following experimental studies. Table S3† shows a comparison of the BPA degradation efficiency of different catalysts. The results indicated that the Co/C-50 catalyst prepared in this study has a competitive advantage in BPA degradation.

#### 3.2.2 Effects of different catalyst dosages

**Fig. 5:**
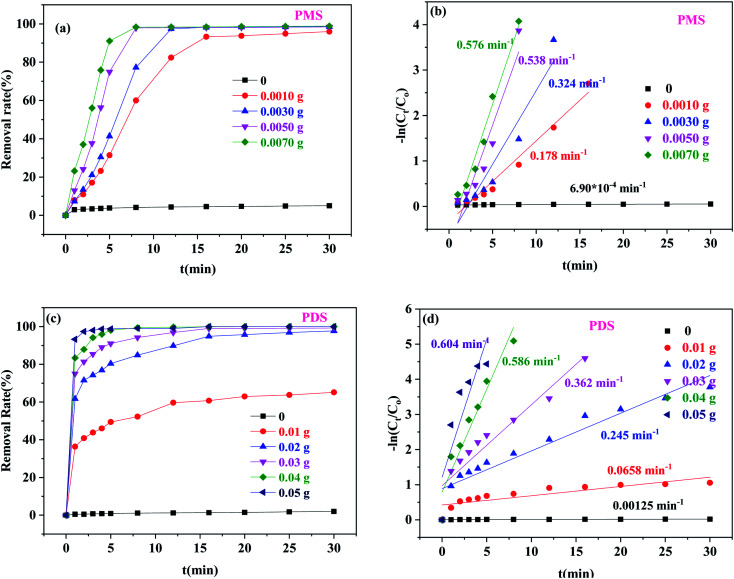
The effects of Co/C-50 dosage on BPA degradation: removal efficiency of BPA with PMS (a) and PDS (c), and corresponding pseudo-first-order kinetic rate constants (b and d) ([PMS] = 0.25 g L^−1^, [PDS] = 1.0 g L^−1^, [BPA] = 20 mg L^−1^, *T* = 30 °C).

Fig. 5 shows the effects of the catalyst dosage on BPA degradation. When the amount of Co/C-50 was 0, the BPA removal efficiencies of PMS and PDS were only 0.95% and 2.02%, respectively. These findings suggested that PMS and PDS alone had little influence on the BPA degradation due to their own oxidation.^38^ In the Co/C-50-PMS system (Fig. 5a and b), the BPA degradation efficiency increased rapidly when the Co/C-50 dosage increased from 0 to 0.0050 g, and the *k*_1_ value increased from 6.90 × 10^−4^ to 0.537 min^−1^. This phenomenon can be attributed to the growth of catalytic active sites, which accelerated the degradation efficiency. However, when the dosage of Co/C-50 increased from 0.0050 g to 0.0070 g, the *k*_1_ value changed from 0.537 min^−1^ to 0.579 min^−1^; the removal efficiency of BPA was not significantly improved due to the delay of mass transfer and unavailability of active sites in the presence of excessive catalyst.^39^ This phenomenon was also shown in the Co/C-50-PDS system (Fig. 5c and d). When the dosage of Co/C-50 was changed to 0.04 g and 0.05 g from 0, the *k*_1_ value increased from 0.00120 min^−1^ to 0.558 min^−1^ and 0.594 min^−1^, respectively. Hence, the optimal Co/C-50 dosages of 0.0050 and 0.0400 g for PMS and PDS activation were chosen for further experiments.

#### 3.2.3 Effect of different oxidant concentrations

**Fig. 6:**
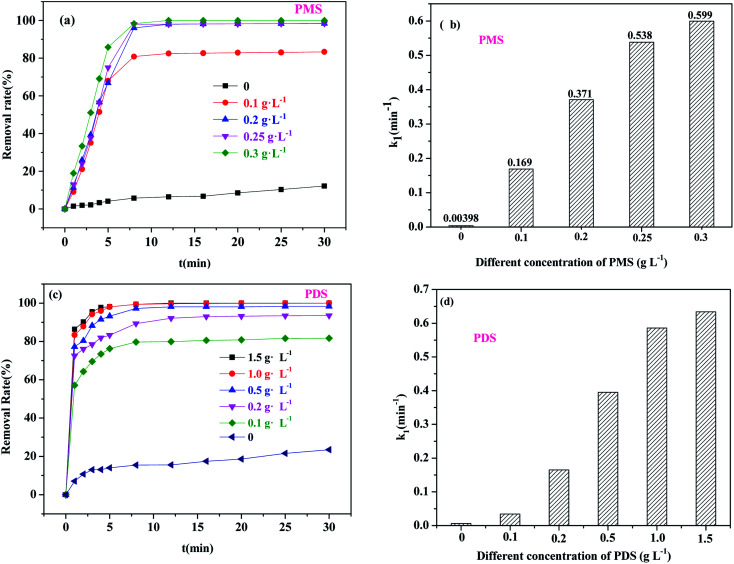
The effects of PS concentration on BPA degradation: (a and c) BPA degradation as a function of time; (b and d) kinetic constants for BPA degradation (PMS system: *m*_catalyst_ = 0.0050 g; PDS system: *m*_catalyst_ = 0.04 g, [BPA] = 20 mg L^−1^, *T* = 30 °C).

Fig. 6 shows the influence of different oxidant concentrations ([PS]_0_) on BPA degradation. As shown in Fig. 6a and b, with the addition of 0 to 0.1 g L^−1^ of PMS, the degradation efficiency of BPA increased rapidly from 7.15% to 80.82%, with the *k*_1_ value increasing from 0.00398 to 0.169 min^−1^; this indicated that the removal of BPA was contributed by oxidative degradation rather than the adsorption of the catalyst. When [PMS]_0_ was changed from 0.1 g L^−1^ to 0.20, 0.25 and 0.30 g L^−1^, the *k*_1_ value increased by 1.20, 2.18 and 2.54 times compared to that of 0.1 g L^−1^ of PMS, respectively. With regard to PDS oxidants, the *k* values of BPA increased nearly linearly with the increase of the PDS concentration (0.1–1.0 g L^−1^), and then the enhancement of the *k* values decreased with further rising of the PDS concentration (Fig. 6c and d). This can be explained by the fact that the excess PDS would react with 
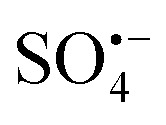
 and ˙OH to reduce the utilization efficiency of PDS^40^ (eqn (2) and (3)). According to the experimental results, the optimal concentrations of PMS and PDS were 0.25 g L^−1^ and 1.0 g L^−1^, respectively.2
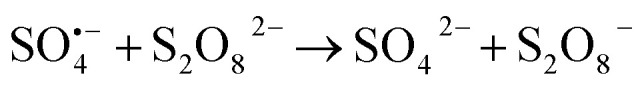

3 ˙OH + S_2_O_8_^2−^ → OH^−^ + S_2_O_8_^−^

#### 3.2.4 Effect of initial pH

**Fig. 7:**
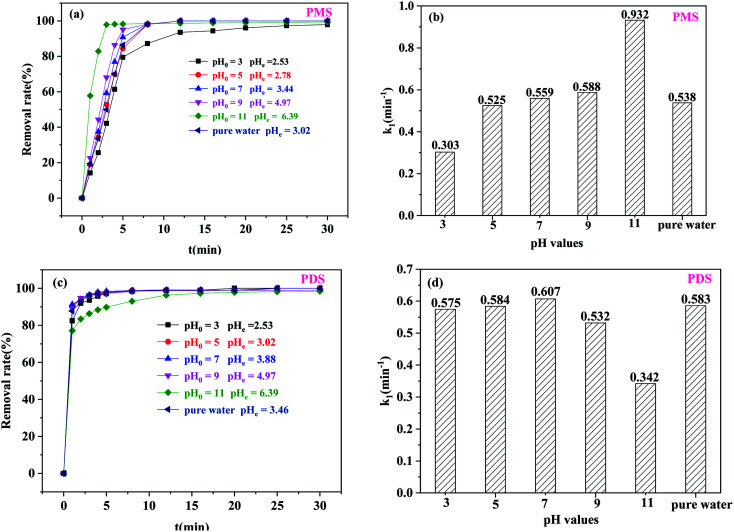
The effect of solution pH: (a and b) PMS system (*m*_catalyst_ = 0.0050 g, [PMS] = 0.25 g L^−1^); (c and d) PDS system (*m*_catalyst_ = 0.04 g, [PDS] = 1.0 g L^−1^) ([BPA] = 20 mg L^−1^, *T* = 30 °C).

Generally speaking, actual water bodies contain a variety of pH values. Therefore, it was very important to evaluate the catalytic activity of the catalysts in the new degradation system to determine whether the organic pollutants could be degraded effectively at various pH values. Therefore, the removal efficiency of BPA was investigated in the Co/C-50-PS system at different initial pH values (pH_0_). When pH_0_ was in the range of 3.0–11.0, BPA could be degraded thoroughly within 30 min (Fig. 7a and b). At higher pH_0_ values, the catalytic degradation efficiency of BPA increased significantly. The *k*_1_ value (0.917 min^−1^) of BPA at pH_0_ = 11 was 3.08 times higher than that (0.298 min^−1^) at pH_0_ = 3. There were five main reasons for this phenomenon: (1) higher leaching of active metal species at lower pH_0_, reducing the active sites on Co/C-50;^40^ (2) the quenching of 
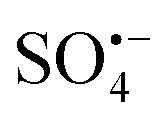
 and ˙OH by H^+^ in acidic conditions (eqn (4) and (5));^41^ (3) activation of PMS by alkali species;^42^ (4) formation of singlet oxygen (^1^O_2_) due to the decomposition of PMS at higher solution pH (eqn (6) and (7));^43^ (5) the increase of the pH value was also conducive to the formation of hydroxyl groups on the surface of the catalyst, which could be used as the active sites for electron transfer, thus improving the performance of the catalyst.^44^ Because PDS had the minimum activation energy^45^ under neutral conditions, BPA had the highest degradation efficiency at pH_0_ = 7, and *k*_1_ reached the maximum value of 0.584 min^−1^ in the Co/C-50-PDS system (Fig. 7c and d).

4 H^+^ + ˙OH + e^−^ → H_2_O

5
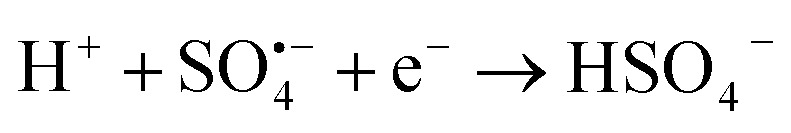

6 HSO_5_^−^ → SO_5_^2−^ + H^+^

7 HSO_5_^−^ + SO_5_^2−^ → SO_4_^2−^ + HSO_4_^−^ + ^1^O_2_

After the reaction, the equilibrium pH (pH_e_) decreased in varying degrees. In the Co/C-50-PMS system (Fig. 7a), the pH_e_ was 2.53, 2.78, 3.44, 4.97 and 6.39 when pH_0_ was set at 3, 5, 7, 9 and 11, respectively. In the Co/C-50-PDS system (Fig. 7c), the pH_e_ decreased to 2.57, 2.74, 3.68, 4.54 and 7.13, respectively. In addition to releasing protons and forming acidic intermediates during BPA decomposition,^25^
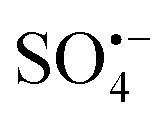
 also reacted with H_2_O to form H^+^ (eqn (8) and (9)).^43^ Moreover, the PMS itself decomposed to yield H^+^ (eqn (10)).^46^8
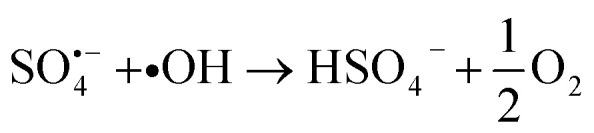

9 HSO_4_^−^ → SO_4_^2−^ + H^+^

10 HSO_5_^−^ → SO_5_^2−^ + H^+^

In general, the Co/C-50-PS system efficiently catalyzed the oxidative removal of BPA under acidic, neutral, and basic conditions, indicating that it has broad application prospects in practical water. The concentration of cobalt ions leached from the reaction solution was determined by ICP-MS. After 30 min reaction in the Co/C-50-PMS system, the concentration of cobalt leaching in the solution was 6.075 mg L^−1^. When the pH value was adjusted to alkaline, almost no cobalt leaching in the solution was observed.

The two systems also exhibited high mineralization efficiency (removal of TOC), which was 75% and 90% in Co/C-50-PMS and Co/C-50-PDS, respectively (Text S5 and Fig. S4†). In the Co/C-50-PS system, hydroquinone and benzoquinone should be the major intermediates of BPA.^47–49^ The ring of benzoquinone could be further cleaved in the presence of 
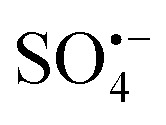
 and ˙OH radicals to form some small molecular organic acids, such as fumaric acid, malonic acid, and formic acid, which should be responsible for the residual TOC.

#### 3.2.5 Effects of reaction temperature and reusability of the Co/C-50 catalyst

The degradation of BPA was fast and efficient under the temperatures of 15 °C, 30 °C and 45 °C, and the fitting reaction activation energies (*E*_a_) were small, which was conducive to trigger the reaction under mild conditions (Text S6 and Fig. S5†). The removal efficiency of BPA remained above 90% after five cycles, exhibiting good reusability of the Co/C-50 catalyst (Text S7 and Fig. S6†).

#### 3.2.6 Effects of the actual water matrix and different target pollutants

Despite the matrix interference, the Co/C-50-PS system exhibited good efficiency for BPA degradation in different actual water samples (Text S8 and Fig. S7†). The results of the Co/C-50-PS system for removal of different pollutants are shown in Text S9 and Fig. S8.†

#### 3.2.7 Free radical quenching experiment and ESR analysis

Previous studies suggested that 
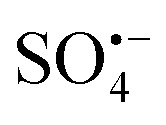
 and ˙OH are the main active substances for the activation of PS by metal-based catalysts.^50^ Consequently, the contribution of free radicals to BPA removal was studied by the free radical quenching method. Methanol (MeOH), as an alcohol containing α-H, has a similar reaction efficiency to ˙OH and 
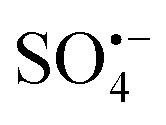
 (eqn (11) and (12)),^51^ and the reaction efficiency of *tert*-butyl alcohol (TBA) without α-H with ˙OH is 3 orders higher than that of 
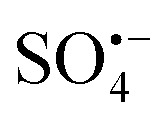
 (eqn (13) and (14)).^52^ Therefore, MeOH was used to quench ˙OH and 
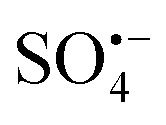
, TBA was used to quench ˙OH and Na_2_S_2_O_3_ was used to quench surface free radicals.

11 CH_3_OH + ˙OH → products, *k* = 9.7 × 10^8^ M^−1^ s^−1^

12
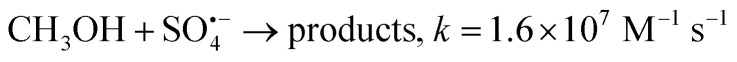

13 TBA + ˙OH → products, *k* = (3.8–7.6) × 10^8^ M^−1^ s^−1^

14
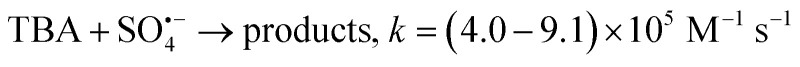


**Fig. 8:**
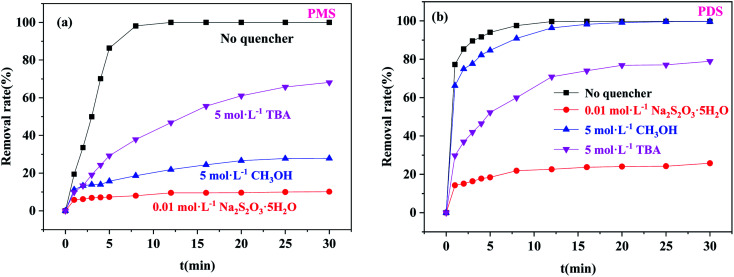
Free radical annihilation experiment: (a) PMS system (*m*_catalyst_ = 0.0050 g, [PMS] = 0.25 g L^−1^); (b) PDS system (*m*_catalyst_ = 0.04 g, [PDS] = 1.0 g L^−1^); ([BPA] = 20 mg L^−1^, *T* = 30 °C).

Fig. 8 shows the degradation efficiency of BPA in pure water, MeOH-aqueous solution, TBA-aqueous solution, and Na_2_S_2_O_3_-aqueous solution. In the Co/C-50-PMS system (Fig. 8a), the removal efficiencies of BPA in pure water, MeOH-aqueous solution, TBA-aqueous solution, and Na_2_S_2_O_3_-aqueous solution were 100%, 27.84%, 68.09%, and 10.14%, and the *k*_1_ values were 0.522, 0.00893, 0.0388, and 0.00225 min^−1^, respectively. Compared with TBA, BPA had a lower degradation efficiency and *k*_1_ value with MeOH addition, which indicated that 
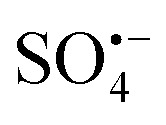
 was the primary active radical. In the Co/C-50-PDS system (Fig. 8b), the removal efficiencies of BPA in pure water, MeOH-aqueous solution, TBA-aqueous solution, and Na_2_S_2_O_3_-aqueous solution were 100%, 100%, 78.97% and 25.78%, respectively, and the corresponding *k*_1_ values were 0.586 min^−1^, 0.313 min^−1^, 0.0684 min^−1^ and 0.00917 min^−1^, respectively. Different from the PMS system, BPA had a lower removal efficiency and *k*_1_ value with TBA addition; this indicated that ˙OH was the dominant radical for BPA degradation, while ˙OH was produced by the reaction of 
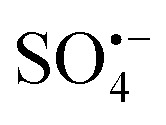
 with H_2_O/OH^−^.^53^

In addition, BPA had the lowest removal efficiency in Co/C-50-PS-Na_2_S_2_O_3_, indicating that surface free radicals played a key role in the degradation process.^50^

**Fig. 9:**
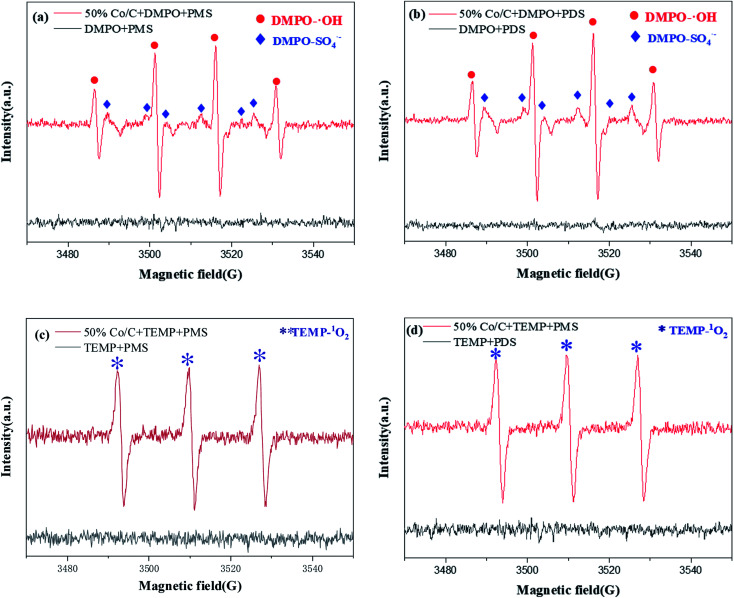
ESR spectra of PMS and PDS activation by Co/C-50. (a) DMPO-˙OH and 
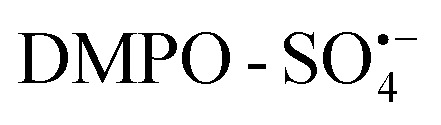
 (PMS); (b) DMPO-˙OH and 
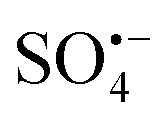
 (PDS); (c) TEMP-^1^O_2_ (PMS); (d) TEMP-^1^O_2_ (PDS) spin adducts (PMS system: *m*_catalyst_ = 0.0050 g, [PMS] = 0.25 g L^−1^; PDS system: *m*_catalyst_ = 0.04 g, [PDS] = 1.0 g L^−1^).

Electron spin resonance (ESR, A300 microx, Bruker, Germany) was used to detect free radicals in the Co/C-50-PS system. DMPO is a spin trapping agent for 
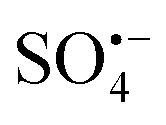
 and ˙OH, and TEMP is a spin trapping agent for ^1^O_2_. No free radical signal was detected when only DMPO was added to the solution containing PS, and DMPO-˙OH and 
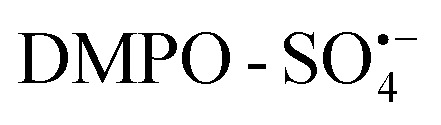
 signal peaks appeared after the Co/C-50 catalyst was added for a period of time (Fig. 9a and b).^54^ Similarly, only TEMP was added to the solution containing PS, and no free radical signal was detected. After adding the Co/C-50 catalyst, the signal peak of TEMP-^1^O_2_ appeared (Fig. 9c and d).^55^ This indicated that Co/C-50 can indeed activate PS to produce 
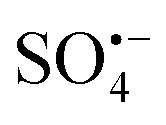
, ˙OH and ^1^O_2_.

## 4. Conclusion

In this study, a Co/C composite was successfully established by one-step activation pyrolysis, and the material exhibited great performance for the degradation and mineralization of BPA by activating PMS and PDS. The reactive oxygen species (ROS) involved in BPA degradation comprised 
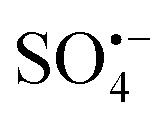
, ˙OH and ^1^O_2_. This new type of cobalt-based heterogeneous catalyst possessed excellent pH adaptability, practical application performance, and reusability for removal of organic pollutants.

## Conflicts of interest

There are no conflicts to declare.

## Supplementary Material

RA-012-D1RA09236G-s001
