# Supplementary material for: Cobalt-loaded cherry core biochar composite as an effective heterogeneous persulfate catalyst for bisphenol A degradation
Source: RSC Adv. 2022 Mar 4;12(12):7284–94. doi: 10.1039/d1ra09236g (PMC8982249; doi:10.1039/d1ra09236g)
Supplement: RA-012-D1RA09236G-s001 [file RA-012-D1RA09236G-s001.pdf]

Electronic Supplementary Material (ESI) for RSC Advances.
This journal is © The Royal Society of Chemistry 2022

Support Information

Cobalt-loaded cherry core biochar composite as effective heterogeneous persulfate catalyst for bisphenol A degradation

Li Li[a], Yuanyuan Zhang[b], Shuangshuang Yang[a], Shengxiao Zhang[a], Qiang Xu[a], Pinzhu Chen[a], Yaxuan Du[a], Yuxin Xing[a]*

[a]School of Chemistry and Materials Science, Collaborative Innovation Center of Shandong Province for High Performance Fibers and Their Composites, Ludong University, Yantai 264025, Shandong province, China

[b]Environmental Monitor Station of Yantai, Shandong Province, China,No. 118, Qingnian South Road, Yantai 264000, Shandong province, China

***Corresponding author: Shengxiao Zhang**

Ludong University

School of Chemistry and Materials Science

Yantai 264025, China

E-mail Address: beijingzsx@163.com

Tel: (086) 0535-6696162; Fax: (086) 0535-6695905

Contents

Text S1 Chemicals and reagents

Text S2 Material characterization

Text S3 Magnetism of material

Text S4 BET characterization of materials

Text S5 Mineralization of BPA

Text S6 Effects of reaction temperature

Text S7 Reusability of Co/C-50 catalyst

Text S8 Effect of actual water matrix

Text S9 Degradation performance for different target pollutants

Table S1 Dosages of raw materials for preparation of different Co/C composites

Table S2 Cobalt content in catalyst

Table S3 Comparison of the degradation efficiency of different catalyst for BPA

Table S4 Water quality conditions of actual water samples

Fig. S1 (a) Hysteresis loop and (b)Magnetic separation behavior of Co/C-50

Fig. S2 FT-IR spectra of different catalysts

Fig. S3 Isotherm Linear Plot of Material

Fig. S4 Total organic carbon analysis: (a) PMS system ($m_{catalyst}$ = 0.0050 g, [PMS] = 0.25 g·L^{-1}); (b) PDS system ($m_{catalyst}$ = 0.04 g, [PDS] = 1.0 g·L^{-1}) ([BPA] = 20 mg·L^{-1}, T = 30 °C)

Fig. S5 Effect of temperature on BPA degradation: (a) PMS system: ($m_{catalyst}$ = 0.0050 g, [PMS] = 0.25 g·L^{-1}); (b) PDS system: ($m_{catalyst}$ = 0.04 g, [PDS] = 1.0 g·L^{-1})

Fig. S6 Reusability tests for BPA removal: (a) PMS system ($m_{catalyst}$=0.0050 g, [PMS]=0.25 g·L^{-1}); (b) PDS system ($m_{catalyst}$=0.04 g, [PDS]=1.0 g·L^{-1}) ([BPA]=20 mg·L^{-1}, T=30 °C)

Fig. S7 Effect of actual water samples on BPA degradation: (a) PMS system: ($m_{catalyst}$ = 0.0050 g, [PMS] = 0.25 g·L^{-1}); (b) PDS system: ($m_{catalyst}$ = 0.04 g, [PDS] = 1.0 g·L^{-1}) (Co/C-50, [BPA] = 20 mg·L^{-1}, T = 30 °C)

Fig.S8 (a, c) pollutants removal as a function of time and (b, d) kinetic constant of different pollutants: (a, b) PMS system ($m_{catalyst}$ = 0.0050 g, [PMS] = 0.25 g·L^{-1}); (b, d) PDS system ($m_{catalyst}$ = 0.04 g, [PDS] = 1.0 g·L^{-1}) ([Pollutants] = 20 mg·L^{-1}, T = 30 °C)

Text S1 Chemicals and reagents

The cherry stones were supplied by a local food processing plant (Yantai,

Shandong Province, China), and then they were ground by ball mill and filtered by 100 mesh sieve. Cobalt chloride hexahydrate ($CoCl_2·6H_2O$), BPA, PDS ($Na_2S_2O_8$), and PMS ($2KHSO_5·KHSO_4·K_2SO_4$) were supplied by Aladdin Biochemical Technology Co. Ltd. (Shanghai, China). Tert-butanol (TBA) was supplied by Tianjin Bodi Chemical Industry (Tianjin, China). HPLC-grade methanol was obtained from Thermo Fisher Scientific Corporation (Shanghai, China). Ultrapure water was produced by using ULUPURE water purification system (Chengdu, Sichuan, China).

Text S2 Material characterization

The surface morphologies of the composite materials were characterized by a high-resolution transmission electron microscope (HR-TEM, Talos F200x, FEI Corp., USA), and the materials were dispersed in ethanol and the mixture was dropped into copper mesh. The elemental composition and chemical valence states of were analyzed using X-ray photoelectron spectroscopy (XPS, Escalab Xi^+, Thermo Scientific, UK) under monochromatic Al Kα radiation, and C 1s peak was used as the internal standard calibration peak at 284.8 eV. Fourier transform infrared (FT-IR) spectra were taken in KBr pressed pellets on a Nicolet iS10 FT-IR Spectrometer (Thermo Fisher Scientific, USA), and analyzed the surface functional groups of the material. The crystal structure of the material was analyzed by X-ray diffractometer (XRD, Ultima IV, Thermo Fisher Scientific, USA) with scanning range of 10-80° and scanning speed of 10°/min. Raman spectrum (in Via reflex, Renishaw PLC, UK) was used to analyze the graphitization degree of the material with an excitation wavelength of 532 cm^{-1} and a test range of 500 - 2500 cm^{-1}.

Text S3 Magnetism of material

The magnetic properties of the materials were analyzed by vibrating sample magnetometer (VSM, BKT-4500). The magnetic properties of Co/C-50 were studied by hysteresis loops, and the results are shown in Fig. S8a. Coercivity of Co/C-50 (27.6Oe) and remanence (2.81 emu/g). Furthermore, the material has a maximum saturation magnetization of 104.0227 emu/g and is large enough to ensure that the catalyst can be easily separated from the solution with magnets. At the same time, the magnetic properties of the prepared materials were proved by Rb-Fe-B magnets (Fig. S8b). Co/C-50 can be completely separated in a few minutes when a Rb-Fe-B magnet is placed next to the reaction solution. If the magnet is removed, the material will disperse again in the solution. Specific magnetic properties facilitate Co/C-50 collection, regeneration and reuse.

Text S4 BET characterization of materials

The specific surface area of the material and biochar was measured using a Micromeritics TriStar II 3020 instrument. According to Eq. S1 and Fig. S2, we can get that the specific surface area of Co/C-50 was 262.5032 m²/g, and that of biochar was 122.6786 m²/g. Co/C-50 had a large specific surface area, which could provide a large number of active sites and promote ion diffusion, which was conducive to the activation of PS and the degradation of organic pollutants.

$$S = \frac{V_m N_A \sigma}{22400W} \quad (V_m = \frac{1}{Slope + intercede}) \tag{S1}$$

Where: NA is the Avogadro constant; σ is the cross-sectional area of each adsorbent molecule; W is the adsorbent mass (g), and 22400 is the volume (mL) of

1mol gas in the standard state.

Text S5 Mineralization of BPA and analysis of metabolite intermediates

The degree of mineralization reflected by the TOC removal rate was another important index to evaluate the performance of the catalyst [1]. The mineralization degree of BPA in Co/C-50-PS catalyst system was quantitatively studied by measuring TOC content. As shown in Fig. S3a, in the system of PMS+BPA, Co/C-50+BPA, and Co/C-50-PMS+BPA, the removal rates of TOC were 6.92%, 16.74%, and 77.76% in 2 h, respectively. Similarly, the TOC removal rate also boosted with time in the Co/C-50-PDS+BPA system (Fig. S3b). After 2 h, the TOC removal rates of PDS+BPA, Co/C-50+BPA and Co/C-50-PDS+BPA were 15.61%, 16.65% and 92.04%, respectively. It presented the same trend as the degradation of BPA, which had the best mineralization effect when both catalyst and PS are present. The results showed that most BPA was oxidized to CO_2 and H_2O finally in Co/C-50-PS system, although some intermediates were generated in the process[2] . The Co/C-50-PS catalyst system not only had good degradation efficiency of BPA, but also had a good mineralization capacity.

Text S6 Effects of reaction temperature

Reaction temperature was a significant factor in the reaction process of AOPs. Three temperature values of 15, 30 and 45 °C were selected to study the effect of temperature on the BPA degradation in Co/C-50-PS system. It could be distinctly seen from Fig. S4 that increasing the reaction temperature could promote the degradation rate of BPA, indicating the reaction was endothermic. In the Co/C-50-

PMS reaction system (Fig. S4a), when the temperature was set from 15 to 45 °C, the k_1 value of BPA degradation increased from 0.325 to 0.793 min^{-1}. Similarly, the k_1 value increased from 0.424 to 0.610 min^{-1} in the Co/C-50-PDS system (Fig. S4b). The principal reasons for the enhancement of reaction rate at high temperature were as follows: (1) Higher temperature could enhance the thermal movement of PS and BPA, especially the transfer of BPA on Co/C-50 surface [3]; (2) Persulfate was more easily activated at elevated temperature [4]. The temperature dependence of the kinetic constants was evaluated by the Arrhenius equation as following (Eq. S2):

$$lnk_1 = -\frac{E_a}{RT} + C \tag{S2}$$

Where k_1 (min^{-1}) represents for the first-order kinetic constant, E_a ($kJ \cdot mol^{-1}$) is reaction activation energy, R (8.314 $J \cdot mol^{-1} \cdot K^{-1}$) and T (K) are ideal gas constant and thermodynamic temperature, and C is constant related to pre-exponential factor. In the light of the illustration, the E_a values of Co/C-50-PMS and Co/C-50-PDS were 22.70 $kJ \cdot mol^{-1}$ (R^2 = 0.9987) and 9.34 $kJ \cdot mol^{-1}$ (R^2 = 0.9896), respectively. The small E_a value predicted the thermodynamic feasibility of the reaction, and 30 °C was enough to provide energy to activate the reaction, so the removal of BPA in Co/C-50-PS system could be carried out under relatively mild conditions.

Text S7 Reusability of Co/C-50 catalyst

Reutilization of heterogeneous catalyst was very significant to enhance water treatment process to become more cost-effective [5]. The magnetic separation and recovery of Co/C-50 material was carried out with an Rb-Fe-B magnet (Text S9, Fig. S8), and then the catalysts were rinsed with $SnCl_2$ solution ($SnCl_2$, as a reducing agent,

can reduce Co^{3+} to Co^{2+}) and pure water for the next experiment. Fig. S5 showed five recycling runs of Co/C-50 catalytic degradation of BPA. The degradation rate of BPA decreased a little after each cycle. In the Co/C-50-PMS catalytic system (Fig. S5a), the removal rates of BPA reduced from 100% (1st) to 98.76% (2nd), 97.21% (3rd), 95.86% (4th), and 92.22% (5th), respectively. In Fig. S5b, the removal rate of BPA in Co/C-50-PDS catalytic decreased from 100% to 91.16% after five cycles. The removal rate of BPA remained above 90% after five cycles. The reasons for the decrease of catalytic activity were as follows: (1) The intermediary products were adsorbed on the surface and covered with some active sites, and the intermediate products would affect the mass transfer between Co/C-50 and PS [6]; (2) Slight loss of the analysts in each recycle reduced the active sites [7]. In general, Co/C-50 had excellent stability and reusability for activation PS to degrade BPA.

Text S8 Effect of actual water matrix

The degradation of BPA by Co/C-50-PS system in ultrapure water, underground water from Dongmotang waterworks (Yantai, Shandong), surface water from Menlou reservoir (Yantai, Shandong), and seawater from Huanghai (Yantai, Shandong) was shown in Fig. S6. The solid impurities in the water sample were removed by pre-treatment through static and filtration operations. At the same time, water quality conditions of several water samples were tested (Table S4). The complex water environment of groundwater and surface water of Dongmatang water plant limited the degradation of BPA by Co/C-50-PS system, which made the removal efficiency of BPA decrease a trifle. The reasons mainly included two aspects: (1) There were other

organic pollutants in actual water, which would compete with BPA; (2) Carbonate species (HCO_3^-, CO_3^{2-}) and phosphate species (HPO_4^{2-}, PO_4^{3-}, $H_2PO_4^-$) existed in actual water samples would quench active radicals [8]. Although the degradation efficiency of BPA in real water sample decreased a little, the removal efficiency still reached almost 100%. Interestingly, because a large amount of Cl^- in seawater could effectively activate PMS but not activate PDS [2,9], the degradation performance of BPA in seawater appeared different phenomenon in Co/C-50-PMS and Co/C-50-PDS. The degradation rate in Co/C-50-PMS could reach 93.71% in 1 min, and attained 100% in 5 min, and seawater ranked the highest reaction rate among these actual water samples. While the degradation efficiency in Co/C-50-PDS showed conspicuous drops, and it was only 48.82% and 74.84% in 1 and 5 min, exhibiting lowest reaction rate. Fortunately, the final removal rate could still reach 96.04% in 30 min. This result indicated that Co/C-50-PS system still owned satisfactory degradation performance for BPA in actual water sample.

Text S9 Degradation performance for different target pollutants

Beside BPA, the removal performance of other refractory organic contaminants such as ibuprofen (IBP), phenol, sunset yellow (SY), and rhodamine B (RhB) was studied to investigate the versatility of Co/C-50-PS catalytic system. In Fig. S7(a, b), when the Co/C-50-PMS system was used, RhB and SY were completely degraded at 3 min and 4 min, and k_1 values were 2.04 and 1.15 min^{-1}, respectively, while the removal efficiencies of BPA, IBP, and phenol were 100%, 92.22%, and 92.58% after 30 min, and the k_1 values were 0.538 min^{-1}, 0.212 min^{-1} and 0.170 min^{-1}, respectively.

Although Co/C-50-PDS system was not as good as Co/C-50-PMS system in degrading five organic contaminants (Fig. S7(c, d)), the removal efficiencies of five kinds of organics still attained 100% (BPA), 80.88% (IBP), 94.55% (phenol), 79.25% (SY), and 99.08% (RhB) after 30 min reaction, and the corresponding k_1 values were 0.586, 0.143, 0.258, 0.0789, and 0.486 min^{-1}, respectively. The diverse removal efficiencies of diverse pollutants may be due to the different molecular architecture [10], for example, –OH and $–CH_3$ groups in BPA make it vulnerable to be attacked by free radical, and heteroatoms in RhB is apt to be oxidized by the electrophilic attack [11], while the low removal efficiency of phenol resulted from its relatively stable structure containing the delocalized π_7^8 bond [12]. The results indicated that the Co/C-50-PS system could be used as a general method for degradation of various organic pollutants.

Table S1 Dosages of raw materials for preparation of different Co/C composites

Name	Mass ratios of Co (%)	$CoCl_2 \cdot 6H_2O$ (g)	cherry stone powder (g)

Activated carbon	0	0	1
Co/C-10	10	0.4037	0.9
Co/C-20	20	0.8075	0.8
Co/C-30	30	1.2112	0.7
Co/C-40	40	1.6150	0.6
Co/C-50	50	2.0187	0.5
Co/C-60	60	2.4224	0.4
Co/C-70	70	2.8262	0.3
Co/C-80	80	3.2300	0.2
Co/C-90	90	3.6337	0.1

Table S2 Cobalt content in catalyst (The amount of catalyst used here is 1.0g/L)

Name	Co/C-10	Co/C-30	Co/C-50	Co/C-70	Co/C-90
**Co(g/L)**	0.1037	0.2994	0.5006	0.7989	0.9008
**Mass ratios of Co/C**	10.37%	29.94%	50.06%	79.89%	90.08%

Table S3 Comparison of the degradation efficiency of different catalyst for BPA

Catalyst	BPA concentration	Catalyst dosage	Oxidant dosage	Removal efficiency	TOC (%)	Refs.
**Fe_3O_4@β-CD/rGO**	20 mg/L	0.5 g/L	10mM (H_2O_2)	100% (30min)	75.9 ± 2.5	[13]
**CoFe/NC**	50 μM	0.1 g/L	0.5 mM (PS)	97% (30min)	85	[14]
**$CoFe_2O_4$**	45 μM	0.1 g/L	0.4mM (PMS)	97% (40min)	81.57	[15]

**Co_3O_4/MXene**	20 mg/L	0.1 g/L	0.3 g/L (PMS)	95% (7min)	31%	[16]
**Co_3O_4/C-BC**	20 mg/L	0.3 g/L	1.0 mM (PMS)	100% (30min)	49.5 ± 4.6	[17]
**Nano-NiO-ZnO**	10 mg/L	50 mg/L	0.1mM (PMS)	95.26% (30min)	67.11	[18]
**$Mn_{0.6}Zn_{0.4}Fe_2O_4$**	0.2 mM	0.2 g/L	0.5 mM (PMS)	95.8% (60min)	70.4	[19]
**Co/C-50**	20 mg/L	50 mg/L	0.25 g/L(PMS)	~100% (8min)	54.13%	This work
		0.4 g/L	1.0 g/L (PDS)	~100% (10min)	90.76%	

Table S4 Water quality conditions of actual water samples

Water sample	Groundwater	Surface water	Seawater
**Na^+ (mg/L)**	41.71	75.44	9457.00
**Ca^{2+} (mg/L)**	62.39	89.83	379.83
**Mg^{2+} (mg/L)**	15.68	39.61	1098.54

**K^+ (mg/L)**	18.76	23.74	363.08
**Cl^- (mg/L)**	55.80	97.87	17328.09
**NO_3^- (mg/L)**	12.00	21.58	0.215
**SO_4^{2-} (mg/L)**	120.00	149.36	2439.74
**F^- (mg/L)**	0.40	0.46	0.73
**pH**	7.4	7.3	8.0
**COD_{Mn} (mg/L)**	0.9	2.8	1.56

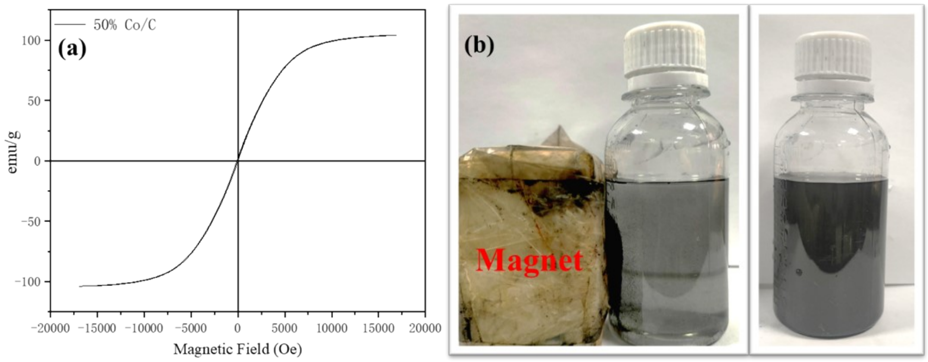

Fig. S1 (a) Hysteresis loop and (b)Magnetic separation behavior of Co/C-50

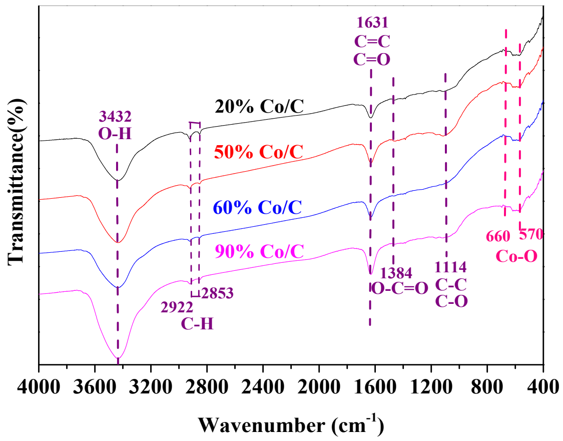

Fig. S2 FT-IR spectra of different catalysts

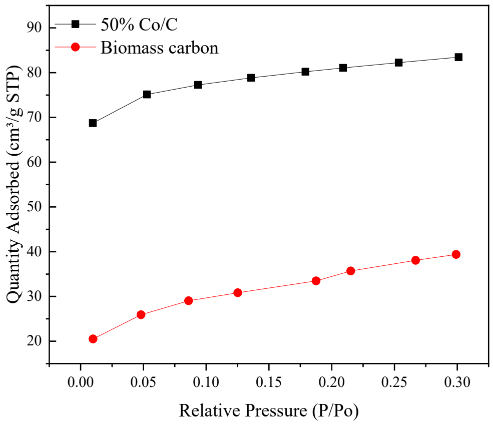

Fig. S3 Isotherm Linear Plot of Material

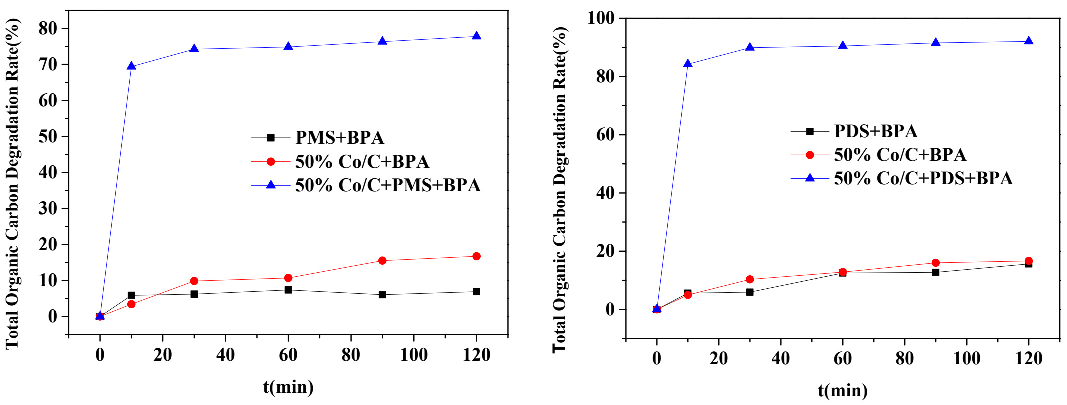

Fig. S4 Total organic carbon analysis: (a) PMS system ($m_{catalyst}$ = 0.0050 g, [PMS] = 0.25 g·L^{-1}); (b) PDS system ($m_{catalyst}$ = 0.04 g, [PDS] = 1.0 g·L^{-1}) ([BPA] = 20 mg·L^{-1}, T = 30 °C)

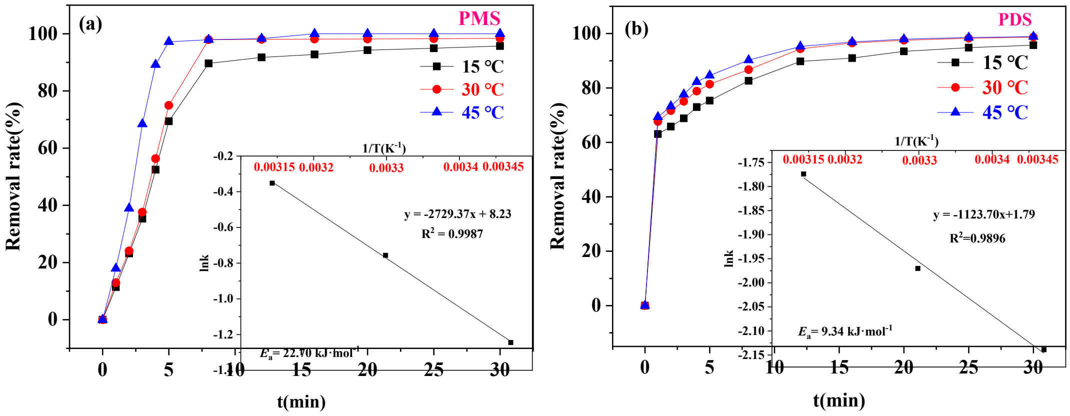

Fig. S5 Effect of temperature on BPA degradation: (a) PMS system: ($m_{catalyst}$ = 0.0050 g, [PMS] = 0.25 g·L^{-1}); (b) PDS system: ($m_{catalyst}$ = 0.04 g, [PDS] = 1.0 g·L^{-1})

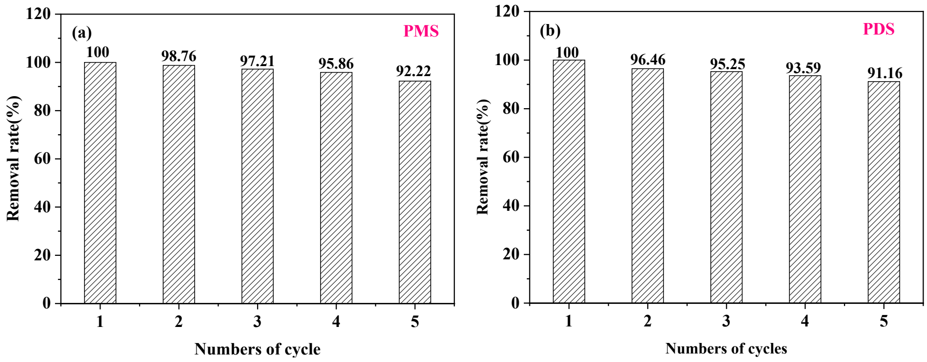

Fig. S6 Reusability tests for BPA removal: (a) PMS system ($m_{catalyst}$=0.0050 g, [PMS]=0.25 g·L^{-1}); (b) PDS system ($m_{catalyst}$=0.04 g, [PDS]=1.0 g·L^{-1}) ([BPA]=20 mg·L^{-1}, T=30 °C)

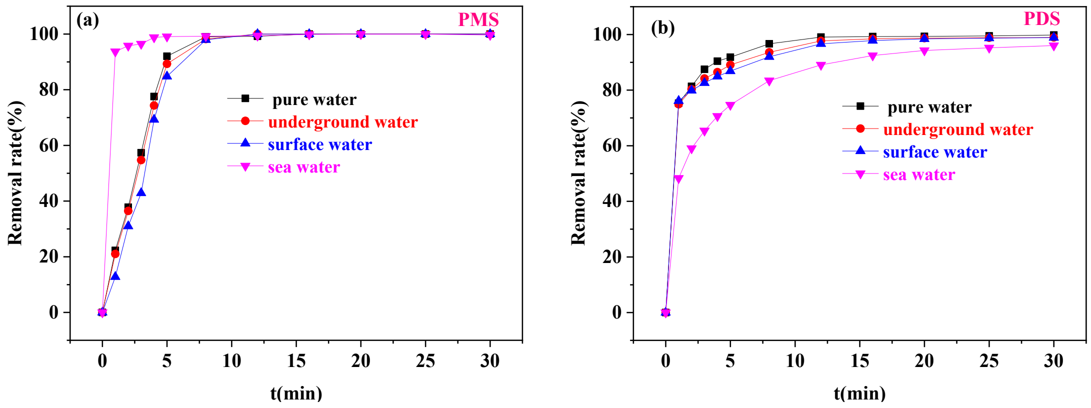

Fig. S7 Effect of actual water samples on BPA degradation: (a) PMS system: ($m_{catalyst}$ = 0.0050 g, [PMS] = 0.25 g·L^{-1}); (b) PDS system: ($m_{catalyst}$ = 0.04 g, [PDS] = 1.0 g·L^{-1}) (Co/C-50, [BPA] = 20 mg·L^{-1}, T = 30 °C)

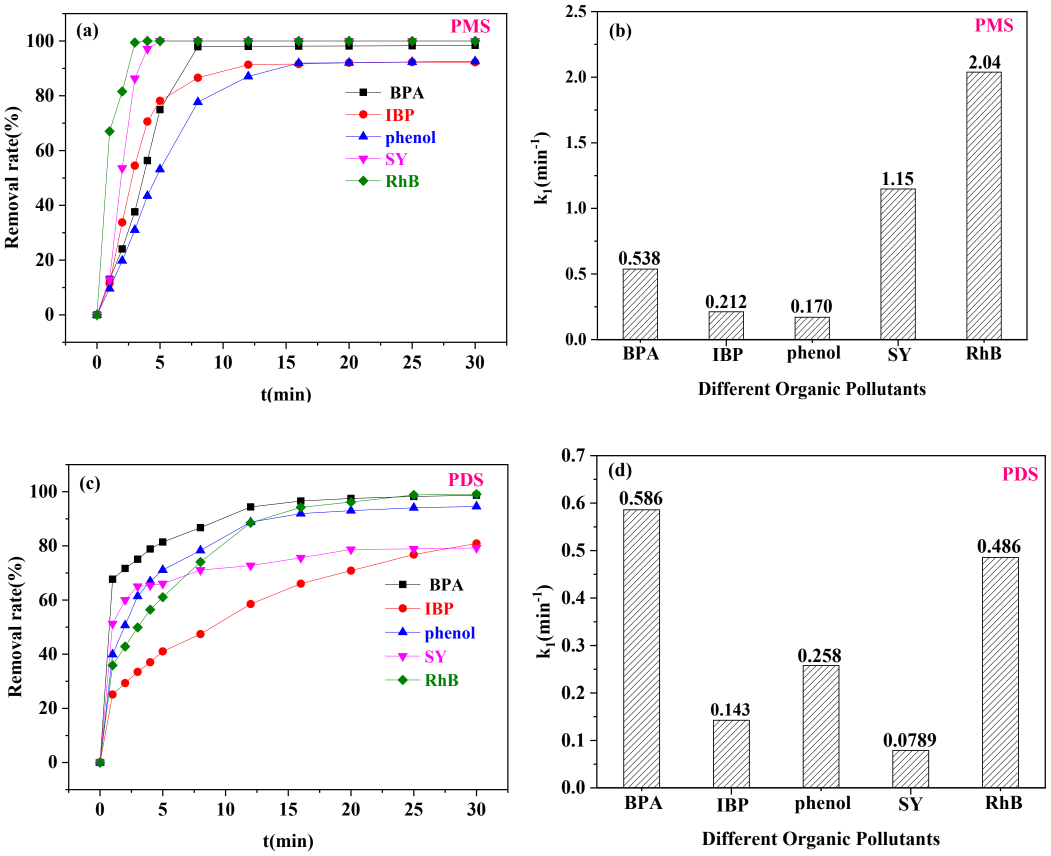

Fig.S8 (a, c) pollutants removal as a function of time and (b, d) kinetic constant of different pollutants: (a, b) PMS system ($m_{catalyst}$ = 0.0050 g, [PMS] = 0.25 g·L^{-1}); (b, d) PDS system ($m_{catalyst}$ = 0.04 g, [PDS] = 1.0 g·L^{-1}) ([Pollutants] = 20 mg·L^{-1}, T = 30 °C)

[1] Zeng T, Li S, Hua J, He Z, Zhang X, Feng H, Song S. Synergistically enhancing Fenton-like

degradation of organics by in situ transformation from Fe3O4 microspheres to mesoporous Fe, N-dual doped carbon. Sci Total Environ, 2018, 645: 550-559.
[2] Yang S, Wang P, Yang X, Shan L, Zhang W, Shao X, Niu R. Degradation efficiencies of azo dye Acid Orange 7 by the interaction of heat, UV and anions with common oxidants: persulfate, peroxymonosulfate and hydrogen peroxide. Journal of Hazardous Materials, 2010, 179(1-3): 552-8.
[3] Gong F, Wang L, Li D, Zhou F, Yao Y, Lu W, Huang S, Chen W. An effective heterogeneous iron-based catalyst to activate peroxymonosulfate for organic contaminants removal. Chem Eng J, 2015, 267: 102-110.
[4] Hussain I, Zhang Y, Huang S. Degradation of aniline with zero-valent iron as an activator of persulfate in aqueous solution. RSC Advances, 2014, 4(7): 3502-3511.
[5] Du J, Bao J, Liu Y, Kim S H, Dionysiou D D. Facile preparation of porous Mn/Fe_3O_4 cubes as peroxymonosulfate activating catalyst for effective bisphenol A degradation. Chem Eng J, 2019, 376.
[6] Qiu X, Yang S, Dzakpasu M, Li X, Ding D, Jin P, Chen R, Zhang Q, Wang X C. Attenuation of BPA degradation by $SO4^{\cdot-}$ in a system of peroxymonosulfate coupled with Mn/Fe MOF-templated catalysts and its synergism with Cl^- and bicarbonate. Chem Eng J, 2019, 372: 605-615.
[7] Liu F, Zhou H, Pan Z, Liu Y, Yao G, Guo Y, Lai B. Degradation of sulfamethoxazole by cobalt-nickel powder composite catalyst coupled with peroxymonosulfate: Performance, degradation pathways and mechanistic consideration. J Hazard Mater, 2020, 400: 123322.
[8] Lescano M R, Lopez A O, Romero R L, Zalazar C S. Degradation of chlorpyrifos formulation in water by the UV/H_2O_2 process: Lumped kinetic modelling of total organic carbon removal. J Photoch Photobio A, 2021, 404.
[9] Wang J, Wang S. Activation of persulfate (PS) and peroxymonosulfate (PMS) and application for the degradation of emerging contaminants. Chem Eng J, 2018, 334: 1502-1517.
[10] Yao Y, Chen H, Lian C, Wei F, Zhang D, Wu G, Chen B, Wang S. Fe, Co, Ni nanocrystals encapsulated in nitrogen-doped carbon nanotubes as Fenton-like catalysts for organic pollutant removal. J Hazard Mater, 2016, 314: 129-139.
[11] Du X, Bai X, Xu L, Yang L, Jin P. Visible-light activation of persulfate by TiO2/g-C3N4 photocatalyst toward efficient degradation of micropollutants. Chem Eng J, 2020, 384: 123245.
[12] Yang L, Bai X, Shi J, Du X, Xu L, Jin P. Quasi-full-visible-light absorption by D35-TiO2/g-C3N4 for synergistic persulfate activation towards efficient photodegradation of micropollutants. Appl Catal B-Environ, 2019, 256: 117759.
[13] Zhang Y, Chen Z, Zhou L, Wu P, Zhao Y, Lai Y, Wang F. Heterogeneous Fenton degradation of bisphenol A using Fe3O4@beta-CD/rGO composite: Synergistic effect, principle and way of degradation. Environ Pollut, 2019, 244: 93-101.
[14] Wang Y, Gao C-Y, Zhang Y-Z, Leung M K H, Liu J-W, Huang S-Z, Liu G-L, Li J-F, Zhao H-Z. Bimetal-organic framework derived CoFe/NC porous hybrid nanorods as high-performance persulfate activators for bisphenol a degradation. Chem Eng J, 2021, 421.
[15] Long X, Yang S, Qiu X, Ding D, Feng C, Chen R, Jihuatan, Wang X, Chen N, Lei Q. Heterogeneous activation of peroxymonosulfate for bisphenol A degradation using CoFe2O4 derived by hybrid cobalt-ion hexacyanoferrate nanoparticles. Chem Eng J, 2021, 404.
[16] Liu Y, Luo R, Li Y, Qi J, Wang C, Li J, Sun X, Wang L. Sandwich-like Co3O4/MXene composite with enhanced catalytic performance for Bisphenol A degradation. Chem Eng J, 2018, 347: 731-740.
[17] Chen X L, Li F, Zhang M, Liu B, Chen H, Wang H. Highly dispersed and stabilized Co3O4/C anchored on porous biochar for bisphenol A degradation by sulfate radical advanced oxidation

process. Sci Total Environ, 2021, 777: 145794.
[18] You J, Sun W, Su S, Ao Z, Liu C, Yao G, Lai B. Degradation of bisphenol A by peroxymonosulfate activated with oxygen vacancy modified nano-NiO-ZnO composite oxides: A typical surface-bound radical system. Chem Eng J, 2020, 400.
[19] Lin H, Li S, Deng B, Tan W, Li R, Xu Y, Zhang H. Degradation of bisphenol A by activating peroxymonosulfate with Mn0.6Zn0.4Fe2O4 fabricated from spent Zn-Mn alkaline batteries. Chem Eng J, 2019, 364: 541-551.
